# Population and molecular datasets for *Gymnadenia conopsea* (Orchidaceae)

**DOI:** 10.1016/j.dib.2019.104161

**Published:** 2019-06-21

**Authors:** Olga E. Valuyskikh, Dmitry M. Shadrin

**Affiliations:** Institute of Biology, Komi Scientific Center, Ural Branch, Russian Academy of Sciences, Syktyvkar, 167982, Russia

## Abstract

The paper presents data on the ecological and phytocoenotic conditions of habitats of the *G. conopsea* (L.) R. Br. orchid in the Northeast of European Russia (Komi Republic, Russia). The data include characteristics of the populations of this species on the northern border of its range (size and ontogenetic structure of the populations, density of specimens, phenology), as well as information demonstrating the genetic variability of the species by ISSR-markers that was not included in the main publication.

The data presented here supplement our earlier published results O.E.Valuyskikh et al., 2019 and are useful for more detailed analysis of population biology and genetic variability of this rare orchid species.

*G. conopsea* is the species of terrestrial orchids widespread in Europe and Asia and characterized by the widest ecological-cenotic amplitude and occurrence in different types of ecotopes. The size of *G. conopsea* populations in different parts of its range is usually small, 25–100 ind. but sometimes increases to 200–1000 ind. Hansen et al., 1999 to Travnichek et al., 2012. In the many regions of the Russian Federation, the *G. conopsea* are subject to protection due to the small number of habitats, long stages of ontogenesis, low population sizes and anthropogenic impact. The complex of *G. conopsea* s.l. included in the Red Data Book of the Komi Republic Taskaev, 2009.

**Table:** Specifications table [*Please fill in right-hand column of the table below.*]

Subject area	*Biology*
More specific subject area	*Botany, Population biology, Genetics*
Type of data	*Table, image, graph, text file*
How data was acquired	*The PCR amplification was carried out on a T100 Thermal Cycler (Bio-Rad, USA).* *Visualization of the PCR products was performed using a UVT-1 transilluminator (Biocom, Moscow).* *The amount of the obtained DNA and PCR products was checked on a Fluorat-02-Panorama spectrofluorometer (Lumex, Russia).* *Sequencing was carried out using ABI Prism BigDye Terminator v.1.1 reagents on an ABI PRISM 310 Genetic Analyzer (Applied Biosystems, USA) at the Center for Collective Use ‘Molecular Biology’ at the Institute of Biology, Komi Scientific Center UrD RAS.*
Data format	*Raw, analyzed*
Experimental factors	*The population observations were performed in the summer of 2005-2007 and 2016–2017. The data were collected from 10 natural populations in different ecotopes that differ in environmental conditions. Samples for genetic analysis were collected in July of 2017. In total, 200 samples (leaves) were collected.*
Experimental features	*The ISSR markers were used.*
Data source location	*Location: Eastern Europe; Country: Russia* *Latitude: 61°N to 62°N* *Longitude: 50°E to 55°E*
Data accessibility	*Data available within the article*[1]
Related research article	*O.E. Valuyskikh, D.M. Shadrin, Ya.I. Pylina, Morphological Variation and Genetic Diversity of Gymnadenia conopsea (L.) R. Br. (Orchidaceae) Populations in the Northeast of European Russia, Russ. J. Genet., 55 (2019) 180–196.*

**Table:** 

**Value of the data** • The presented data can be used by other researchers to compare the habitats, phenology and population genetic characteristics of the G. conopsea orchid in different parts of the range and to develop methods for the protection of this rare species. • The raw data obtained from the ISSR analysis results will allow other researchers to expand the statistical analysis in this field. • A graphical representation of the results of data analysis using the Structure 2.3 program demonstrates the algorithm for selecting the number of groups (K) in the sample selection under study for different models [1].

## 1 Data

**Fig. 1:**
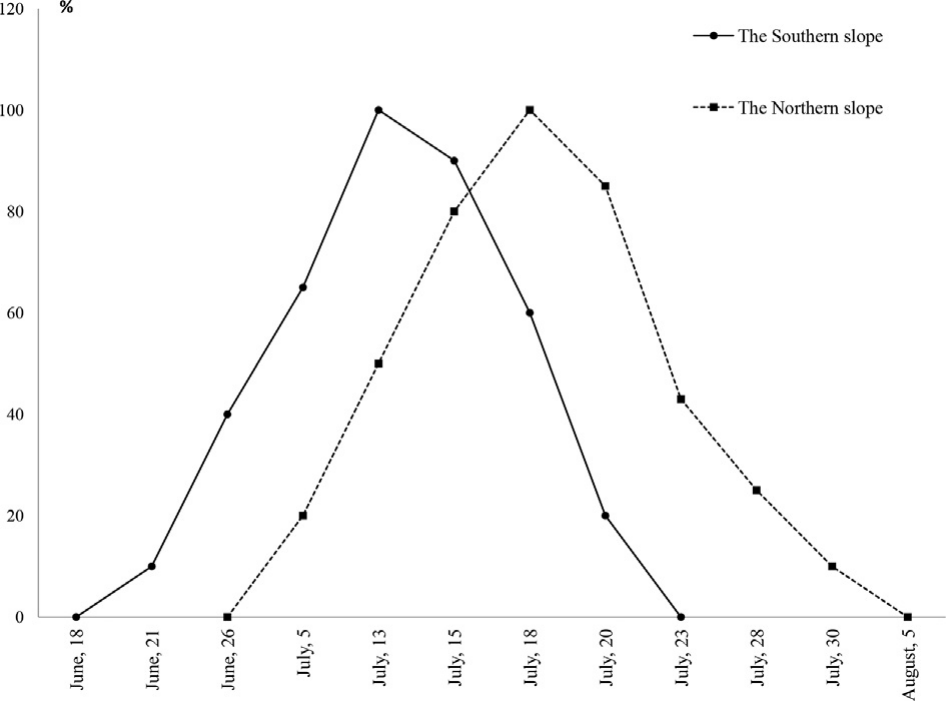
The share of blooming *G. conopsea* plants in populations on the slopes with limestone outcrops of different orientations.

The paper presents data supplementing our study of the morphological variability and genetic diversity of *G. conopsea* populations in the North-East of European Russia [1]. The fragrant orchid *G. conopsea* growing in the Komi Republic, near its northern distribution limit, where it occurs mainly on limestone outcrops in river valleys [7]. A comparative analysis of cenopopulations from different parts of the range has shown that their structure is often independent of geographic location but is determined mainly by ecocenotic conditions in habitats [2], [3], [4], [5], [6]. The obtained data reflects the ecological and coenotic conditions of the habitats of this species in the middle taiga subzone on limestone outcrops of the Timan Ridge (9 populations) and on the Mezen-Vychegda Plain (1 population). A brief description of the communities where the species can be found is provided in Table 1. It should be noted that species with arctic, arctic-alpine and subarctic types of habitats grow on the “cold” slopes, whereas forest steppe and pine forest species grow on the “warm” slopes. Such qualitative parameters of *G. conopsea* populations as size, density, shares of specimens at different ontogenetic stages, restoration index are provided in Table 1. The *G. conopsea* populations located in different ecotopes have different dates of beginning of phenological phases, *e***.***g***.** blooming (Fig. 1).

**Table 1:** Sample collection sites and characteristics of *G. conopsea* populations.

No	Code of the population*	Location	Ecologic-cenotic conditions	Coordinates	Characteristics of the populations								
					Number, units	Study year	Density, inds./m^2^	Ontogenetic spectrum, %					Iϑ
								*j*	*im*	*v*_1_	*v*_2_	*g*	
1	N1	South Timan, left bank of the Soiva River. Middle part of the northwestern slope, inclination 40°–45°.	Spruce open stoneberry-moss forest. The crown density is 0.2–0.3. The tree layer consists of *Betula pendula* Roth*, Pinus sylvestris* L*.* The grasses and dwarf shrubs (total projective cover 25%) are dominated by *Rubus saxatilis* L. *Vaccinium uliginosum* L*., Saussurea alpina* (L.) DC., *Dryas octopetala* L., *Tephroseris integrarifolia* (L.) Holub, *Persicaria vivipara* (L.) Ronse Decr*.,* etc. The mossy-lichen cover makes 45–50%.	62°44′995″ 55°50′468″ 161 m a.s.l.	>1000	2005	6.6	18.4	31	32.7	2.0	15.8	5.33
						2006	5.1	23.5	41.2	15.68	0	19.6	4.10
						2007	15.9	11.2	40.5	28.1	2.2	18.0	4.56
						2016	9.7	35.5	34.3	18.3	1.2	10.7	8.4
2	L2	South Timan, left bank of the Soiva River. Low floodplain in the river valley.	Lady's mantle-herb meadow. The total projective cover (TPC) makes 85–95%. The grassy cover includes *Trollius europaeus* L., *Briza media* L., *Sanguisorba officinalis* L., *Persicaria vivipara* (L.) Ronse Decr*.*, *Geranium sylvaticum* L., *Elymus caninus* (L.) L., *Dactylorhiza fuchsii* (Druce) Soó, *Alchemilla* sp., etc. *G. conopsea* is a co-dominant. Green mosses and lichens take 3–4%.	62°45′129″ 55°50′292″ 136 m a.s.l.	200–300	2005	4.5	11.8	5.8	15.7	7.8	58.8	0.70
						2006	6.8	2.9	23.5	29.4	13.2	30.8	2.24
						2007	10.4	8.6	19.2	16.3	6.7	49.0	1.04
						2016	8.9	5.4	10.7	21.4	7.1	55.4	0.8
3	M3	South Timan, left bank of the Soiva River. Low floodplain in the river valley at the foot of the slope.	Herb-grass meadow. The TPC is 80–90%. The grassy layer is dominated by *Anthoxanthum odoratum* L., *Trollius europaeus* L., *Elymus caninus* (L.) L., *Filipendula ulmaria* (L.) Maxim., *Sanguisorba officinalis* L., *Stellaria holostea* L., *Lathyrus pratensis* L., *Antennaria dioica* (L.) Gaertn., *G. conopsea,* etc. The mossy-lichen cover (MLC) is less than 10%.	62°44′880″ 55°49′490″ 135 m a.s.l.	200–300	2005	6.2	5.4	10.8	24.3	6.1	53.5	0.68
						2006	10.4	5.8	20.2	47.2	7.7	19.2	4.20
						2007	7.3	1.3	4.0	5.3	12.0	77.3	0.29
						2016	8.2	2.8	8.5	11.3	2.8	74.7	0.3
4	S4	South Timan, left bank of the Soiva River. Middle part of the southern slope, inclination 45°-50°.	Birch open forest. The crown density is 0.2. Shrubs are represented by *Spiraea media* Schmidt*.* The TPC of grasses and dwarf shrubs is 25–30% dominated by *Rubus saxatilis,* *Festuca ovina* L.*, Aster alpinus* L.*, Lathyrus vernus* (L.) Bernh. Встречаются *Thymus talijevii* Klokov & Des.-Shost., *Epipactis atrorubens* (Hoffm.) Besser*, Dendranthema zawadskii* (Herbich) Tzvel.*, Adonis sibirica* Patrin ex Ledeb.*, Melica nutans* L, etc. The MLC makes 45% and includes *Hylocomium splendens* (Hedw.) Schimp., *Pleurozium schreberi* (Willd. ex Brid.) Mitt., etc.	62°44′768″ 55°49′036″ 150 m a.s.l.	>500	2005	5.4	7.9	33.7	17.6	16.1	24.6	2.78
						2006	11.3	6.2	33.8	31.8	9.7	18.6	4.38
						2007	8.9	19.2	41.3	28.4	5.5	5.5	17.2
						2016	7.6	13.9	25.4	34.8	10.5	15.3	5.1
5	N5	South Timan, right bank of the Soiva River. Middle part of the northern slope, inclination 45°.	*Pinus sylvestris, Picea obovata, Betula pendula* after-growth. Shrubs are represented by *Juniperus communis* L. The TPC of grasses and dwarf shrubs is 5–20% with abundant *Arctostaphylos uva-ursi* (L.) Spreng., *Festuca ovina*, *Vaccinium uliginosum.* Реже *Dryas octopetala, Valeriana capitata* Pall. ex Link.*, T. talijevii*, *Asplenium viride* Huds, *G. conopsea,* etc. The MLC (TPC 80%) includes *Hylocomium splendens*, *Pleurozium schreberi*, *Cladonia* sp., *Cladina* sp.	62°44′773″ 55°49′516″ 176 m a.s.l.	>1000	2005	7.5	27.7	31.3	22.8	2.4	15.7	5.38
						2006	18.1	33.1	48.1	16.0	0	2.8	35.2
						2007	12.9	36.3	47.6	10.1	2.3	3.6	27.0
						2016	11.2	36.8	41.2	13.8	2.1	6.2	15.0
6	M6	South Timan, left bank of the Omra River. Low floodplain in the river valley at the foot of the slope.	Floodplain grass-herb meadow. The TPC is 60–70%. The grass stand counts 33 species dominated by *Lathyrus pratensis* L.*, Galium boreale* L*., G. conopsea, Carex flava* L., *Trifolium pratense* L*., Ligularia sibirica* (L.) Cass., etc. The MLC is well-developed (TPC 30%) and normally consists of green mosses.	62°45′534″ 55°51′840″ 143 m a.s.l.	200–300	2005	8.8	6.8	15.9	7.9	5.6	63.6	0.57
						2006	5.5	0.0	25.9	21.2	3.8	48.1	1.08
						2007	6.1	2.5	12.3	24.0	4.1	56.8	0.76
						2016	4.3	15.6	26.0	11.5	3.1	43.7	1.3
7	N7	South Timan, right bank of the Omra River. Middle part of the northern slope, inclination 40°-45°.	Spruce-pine open forest. The crow density is 0.1. There are young trees of *Picea obovata, Pinus sylvestris, Betula pendula*. The projective cover of shrubs makes 5% and includes *Juniperus communis, Lonicera pallasii* Ledeb*., Salix* sp. The TPC of grasses and dwarf shrubs is 20–25% dominated by *Dryas octopetala*, *Carex glacialis* Mackenz.*, Festuca ovina, Antennaria dioica*, *Aster alpinus, Vaccinium vitis-idaea.* The MLC includes *Hylocomium splendens*, *Pleurozium schreberi,* etc.	62°45′218″ 55°51′931″ 177 m a.s.l.	>1000	2005	8.3	19.6	35.2	25.5	0	19.6	4.10
						2006	7.6	22.6	29.8	33.3	0	13.2	6.55
						2007	9.0	25.0	39.5	25.0	1.3	9.2	9.86
						2016	9.2	21.0	38.3	26.7	1.2	12.8	6.8
8	S8	South Timan, left bank of the Omra River. Middle part of the southwestern slope, inclination 30°.	Spruce grass-lichen-moss open forest. The crown density is 0.1. The tree layer holds *Pinus sylvestris, Betula pendula*. The projective cover of shrubs makes 5% including *Juniperus communis, Lonicera pallasii*. The layer of grasses and dwarf shrubs with the TPC of 20–25% is dominated by *Carex glacialis.* The MLC (TPC 60%) is dominated by *Hylocomium spleendens*, *Pleurozium schreberi*, *Cladonia* sp., *Cladina* sp., *Peltigera* sp., etc.	62°45′263″ 55°52′328″ 181 m a.s.l.	>500	2005	4.5	18.9	24.3	16.6	2.7	37.8	1.64
						2006	5.6	10.2	29.3	35.3	7.3	17.7	4.09
						2007	14.4	39.8	32.5	18.4	3.6	5.5	17.1
						2016	7.7	30.5	30.5	22.1	3.2	13.7	6.3
9	S9	South Timan, left bank of the Omra River. Upper part of the southwestern slope, large fragmental debris, inclination 40°.	Single *Pinus sylvestris* trees. Young trees are *Picea obovata, Betula pendula, Salix* sp*.* The layer of grasses and dwarf shrubs (TPC 15–20%) is dominated by *Vaccinium uliginosum, Dryas octopetala, Arctous alpina*, *Dendranthema zawadskii, Campanula rotundifolia* L*., Parnassia palustris* L., *G. conopsea, Arctostaphylos uva-ursi,* etc. The MLC makes 35–40%.	62°45′112″ 55°52′919″ 140 m a.s.l.	>300	2005	3.8	5.2	30.7	28.2	17.2	17.9	4.57
						2006	7.9	1.4	37.8	29.1	6.3	25.3	2.95
						2007	7.5	7.6	29.3	35.8	19.2	9.1	10.1
						2016	5.6	12.8	35.7	26.3	4.7	20.3	3.9
10	MF10	Mezen-Vychegda Plain.	Marshy spruce bogbean-sedge-hypnum sphagnum forest. The sparse underwood consists of *Salix myrsinifolia* Salisb., *Betula pubescens* Ehrh. The well-developed grassy layer (TPC 80%) is dominated by *Carex cespitosa* L., *Carex rostrata* Stokes, *Equisetum palustre* L., *Persicaria bistorta*, *Caltha palustris* L. The MLC is well-developed (TPC 30%) and normally includes sphagnum mosses (*Sphagnum capillifolium* (Ehrh.) Hedw., *Sphagnum cuspidatum* Ehrh. ex Hoffm.).	61°37′50.5″ 50°40′49.8″ 110 m a.s.l.	<200	2016	2.4	0	9.5	19.1	14.3	57.1	0.75

Note: Populations from the slopes of the northern and northwestern exposure (N1, N5, N7), southern and southwestern exposure (S4, S8, S9), meadows in the river valleys (M2, M3, M6) of the South Timan, and marshy forests from the Mezen-Vychegda Plain (MF10) are described. Keys: Iϑ – restoration index.

**Fig. 2:**
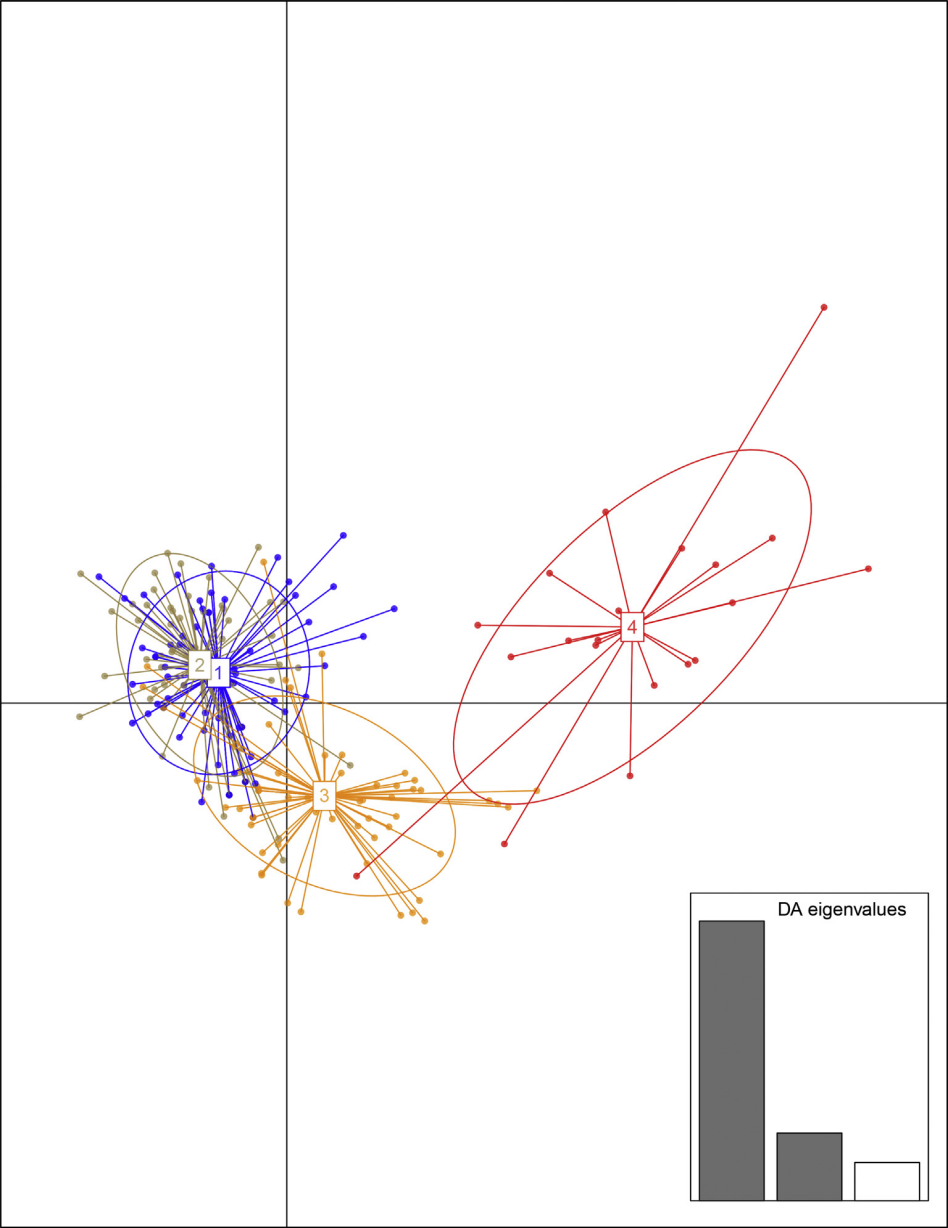
The dispersion diagram plotted from the results of the discriminant analysis of principal components (DAPC) of the matrix of ISSR loci of *G. conopsea* populations (PC = 30): 1 – populations from northern slopes (N1, N5, N7), 2 – populations from southern slopes (S4, S8, S9), 3 - populations from meadows (M2, M3, M6), 4 - population from a boggy forest from the Mezen-Vychegda Plain (MF10).

**Fig. 3:**
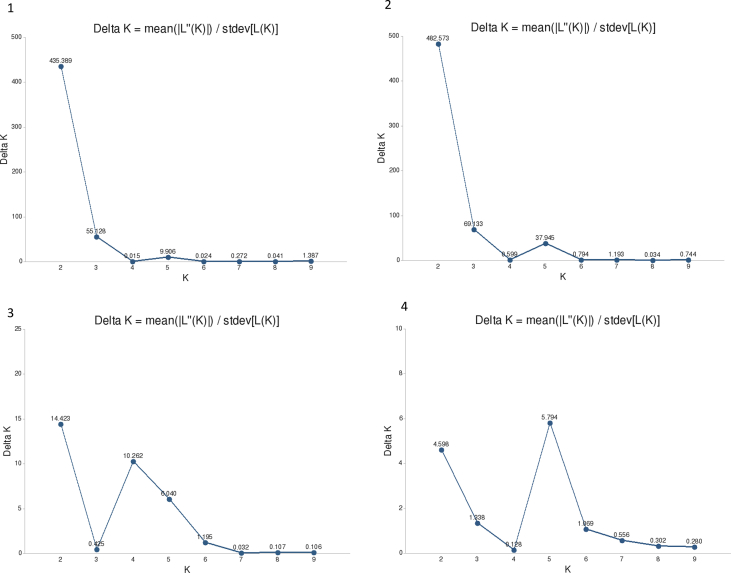
K values used to assess the population structure of *G. conopsea*: 1 — K values for 10 populations using the “admixture” model; 2 — K values for 10 populations using the “no admixture” model; 3 - K values for 9 populations from the South Timan (except for BF10 population) using the “admixture” model; 4 - K values for 9 populations from the South Timan (except for BF10 population) using the “no admixture” model.

The primary matrix of informative ISSR loci for 200 samples from 10 *G. conopsea* populations using two pairs of primers is shown in Table 2. An analysis of the genetic structure of the samples under study performed by the method of discriminant analysis of principal components (DAPC) made it possible to group the sample selections according to their ecotopes (Fig. 2). Fig. 3 shows the graph of dependence of Delta K on K (K is a hypothetical number of isolated genetic groups in the sample selection under study) that is used to assess the population structure with the Structure 2.3 program [8], [9].

**Fig. 4:**
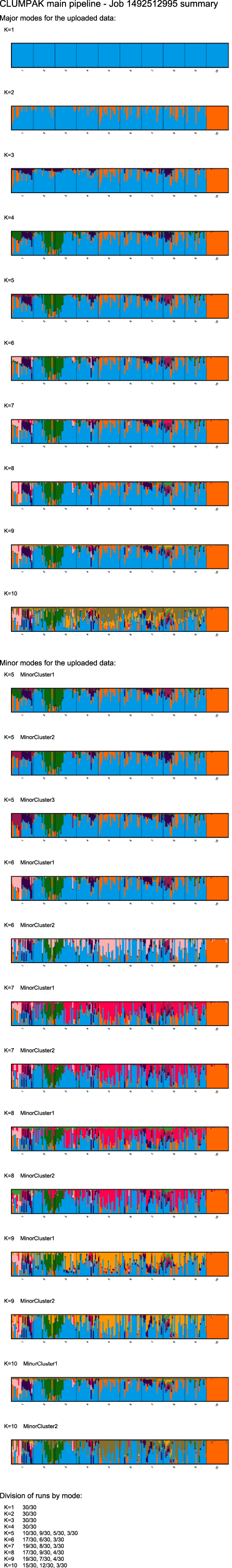
Results of cluster analysis of composition of ISSR loci of *G. conopsea* obtained with the Structure program for all 10 populations using the “admixture” model. The numbers on the X-axis indicate the numbers of populations (see Table 1).

**Fig. 5:**
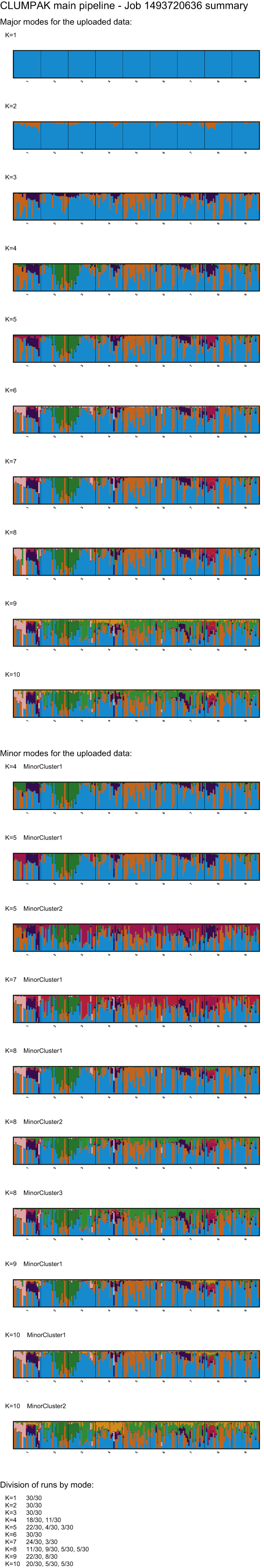
Results of cluster analysis of composition of ISSR-loci of *G. conopsea* obtained with the Structure program for 9 populations using the “admixture” model. The numbers on the X-axis indicate the numbers of populations (see Table 1).

The program allows estimating the genetic structure of populations and the probability of finding each individual as part of a cluster K within which the deviation from Hardy–Weinberg equilibrium would be minimal. The most probable K value (the hypothetical number of isolated genetic groups in the studied sample) was determined by the method proposed in the study of G. Evanno et al. [10]. For a numerical evaluation of the homogeneity (convergence) of the results obtained at independent Structure startups, the CLUMPP version 1.1.2b program [[11], http://clumpak.tau.ac.il/] was used. The obtained graphs (constructed with the DISTRUCT version 1.1 program, https://web.stanford.edu/group/rosenberglab/distruct.html) show the probable population structure for 10 Structure replicates using 200 specimens from 10 to 9 *G. conopsea* populations (Fig. 4, Fig. 5, respectively).

## 2 Experimental design, Materials, and Methods

**Fig. 6:**
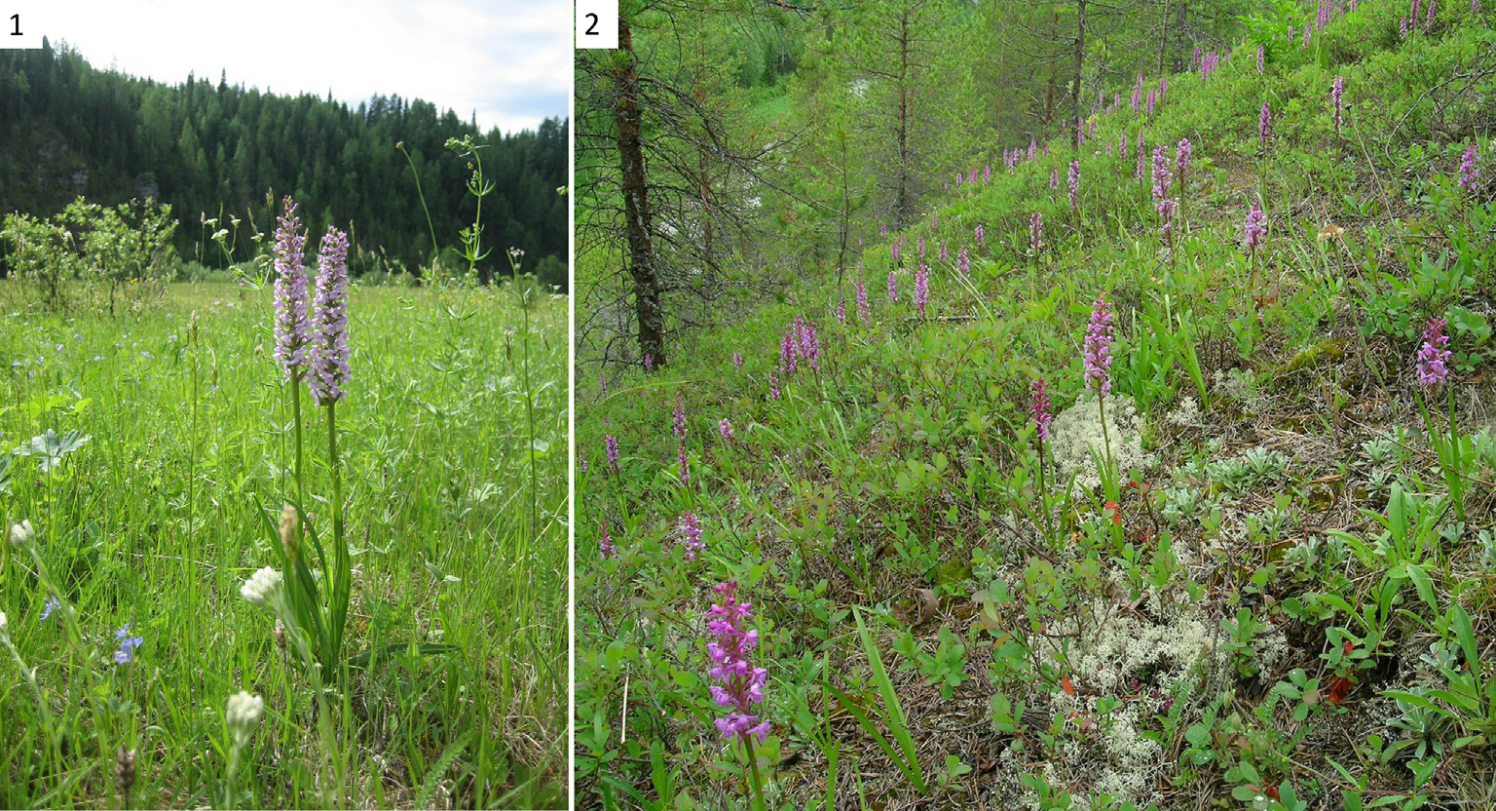
Blooming *G. conopsea* specimens on meadows and forested limestone slopes of the South Timan.

Samples were collected in natural *G. conopsea* populations (2005–2007, 2016) in the North-East of European Russia in different orographic regions: the South Timan Ridge and Mezen-Vychegda Plain. The studied South Timan populations were located in several types of karst landscapes: on the northern and north-western slopes, on the southern and south-western slopes, as well as on the flattened surfaces in the river valleys (Fig. 6). The names of vascular plants are given according to The Plant List (http://www.theplantlist.org/). The plant communities were cut into transects which were divided into counting sites of 1 m^2^ in size with registration of specimens at different ontogenetic stages (*j*, *im*, *v1*, *v2*, *g*). The sites were arranged linearly along a transect 20–30 m long or in parallel as adjacent strips 8–10 m long.

When identifying ontogenetic stages, we used the concept of discrete description of ontogenesis while taking into account the characteristics of individual development of *G. conopsea* [5], [12]. Plants classified as **juvenile** (*j*) have a single middle leaf 2.7–13.5 cm long and 0.1–0.6 cm wide. The belowground sphere of such plants is diverse and may be represented by a protocorm alone, a protocorm and the first thickened adventitious root, or one or two adventitious roots and a root-stem tuberoid with several cordlike endings (lobes). The main diagnostic character for attributing a plant to the **immature** (*im*) group is the presence of two middle leaves 4.5–16 cm long and 0.2–0.9 cm wide. Such plants usually have a root-stem tuberoid with two to four lobes and two to four adventitious roots (rarely, only one root). Our criterion for the **virginile** ontogenetic state is readiness for blooming in the next year, which is characteristic of plants with three to five or six assimilating leaves. We distinguish them into two subgroups, taking into account morphological adaptations of the species to a wide range of ecological conditions in the study region. The “**young**” vegetative subgroup (*v*_1_) comprises plants with three assimilating leaves, tuberoid with two to six lobes, and three to six adventitious roots. The leaves are 5.5–15.5 cm long and 0.3–1.2 cm wide, with 5–9 (11) veins. Plants of the “**adult**” vegetative subgroup (*v*_2_) have four to six leaves, tuberoid with three to nine lobes, and four to eight adventitious roots. The leaves are 6.3–16.5 cm long and 0.4–1.3 cm wide, with 5–10 veins. **Generative** (*g*) plants have a 13.5–51.5 cm tall shoot with 3–6 middle leaves and 3–10 adventitious roots.

The counting unit was a specimen of seed origin. For each population, we determined the number of specimens (units), the mean density of plants (spec./m^2^), the share of specimens at different developmental stages (%), and restoration index presenting the ratio of young specimens to adult ones. The studies of genetic diversity and structure of 200 samples from 10 *G. conopsea* populations were carried out at the Center for Collective Use “Molecular Biology” of the Institute of Biology of the Komi Scientific Center of the Ural Division of the Russian Academy of Sciences (Syktyvkar). The detailed methodology is described in the work [1].

## Appendix A Supplementary data

Table 2. Raw data of the informative ISSR loci for 200 samples from 10 *G. conopsea* populations.
